# Structural correlates of skilled performance on a motor sequence task

**DOI:** 10.3389/fnhum.2012.00289

**Published:** 2012-10-19

**Authors:** Christopher J. Steele, Jan Scholz, Gwenaëlle Douaud, Heidi Johansen-Berg, Virginia B. Penhune

**Affiliations:** ^1^Department of Psychology, Concordia UniversityMontréal, QC, Canada; ^2^Department of Neurology, Max Planck Institute for Human Cognitive and Brain SciencesLeipzig, Germany; ^3^Mouse Imaging Centre, Hospital for Sick ChildrenToronto, ON, Canada; ^4^FMRIB Centre, University of Oxford, John Radcliffe HospitalHeadington, Oxford, UK

**Keywords:** superior longitudinal fasciculus, individual differences, motor sequence performance, fractional anisotropy, diffusion tensor imaging, gray matter volume

## Abstract

The brain regions functionally engaged in motor sequence performance are well-established, but the structural characteristics of these regions and the fiber pathways involved have been less well studied. In addition, relatively few studies have combined multiple magnetic resonance imaging (MRI) and behavioral performance measures in the same sample. Therefore, the current study used diffusion tensor imaging (DTI), probabilistic tractography, and voxel-based morphometry (VBM) to determine the structural correlates of skilled motor performance. Further, we compared these findings with fMRI results in the same sample. We correlated final performance and rate of improvement measures on a temporal motor sequence task (TMST) with skeletonized fractional anisotropy (FA) and whole brain gray matter (GM) volume. Final synchronization performance was negatively correlated with FA in white matter (WM) underlying bilateral sensorimotor cortex—an effect that was mediated by a positive correlation with radial diffusivity. Multi-fiber tractography indicated that this region contained crossing fibers from the corticospinal tract (CST) and superior longitudinal fasciculus (SLF). The identified SLF pathway linked parietal and auditory cortical regions that have been shown to be functionally engaged in this task. Thus, we hypothesize that enhanced synchronization performance on this task may be related to greater fiber integrity of the SLF. Rate of improvement on synchronization was positively correlated with GM volume in cerebellar lobules HVI and V—regions that showed training-related decreases in activity in the same sample. Taken together, our results link individual differences in brain structure and function to motor sequence performance on the same task. Further, our study illustrates the utility of using multiple MR measures and analysis techniques to specify the interpretation of structural findings.

## Introduction

Even with identical practice, no two individuals are able to reach the same level of performance on a motor skill—nor do they follow the same trajectory of improvement as they learn. As neuroscientists, we assume that such individual differences are related to brain structure and function, but relatively few studies have linked performance variability to variability in the brain. Over the last 20 years, work with animals and functional neuroimaging studies in humans have identified the major brain regions involved in learning and performing motor skills (Hikosaka et al., 2002; Doyon and Benali, 2005; Ashe et al., 2006; Doyon et al., 2009; Penhune and Steele, 2012). Work from our lab and that of others has examined the relationship between individual differences in motor performance and brain function (Seidler et al., 2002; Penhune and Doyon, 2005; Grafton et al., 2008; Seidler and Noll, 2008; Orban et al., 2010; Steele and Penhune, 2010), but individual differences in structure have rarely been explored (Van Horn et al., 2008). However, recent studies have shown that individual differences in white matter (WM) supporting visuospatial attention (Tuch et al., 2005), motor cortical connectivity through the corpus callosum (Johansen-Berg et al., 2007), and connectivity between the motor regions of the cerebellum and motor cortex (Della-Maggiore et al., 2009; Tomassini et al., 2011) can be related to motor performance. Only one of those studies combined measures of WM and gray matter (GM) structure with functional MRI (Tomassini et al., 2011). Crucially, the authors found adjacent functional- and WM-performance correlations in the dorsal premotor region, and WM-performance correlations that were adjacent to co-localized functional- and GM-correlations in cerebellar crus I–II and lobule HVI (Tomassini et al., 2011). These results underscore the importance of combining data from multiple methodologies to provide a more nuanced view of how brain structure and function are related to behavior. Following this model, the current study combines fMRI data from a study of motor sequence learning (Steele and Penhune, 2010) with diffusion tensor imaging (DTI—to assess WM integrity and perform probabilistic tractography) and voxel-based morphometry (VBM—to assess GM volume). The goal is to examine the relationship between individual differences in performance, brain function, and underlying structure at the end of training. Results from the fMRI experiment revealed learning- and performance-related functional changes in motor, cerebellar, and parietal cortex. Based on this, we hypothesized that individual differences in WM and GM structure in these regions would be related to individual differences in motor sequence performance.

The majority of structural studies of individual differences find that better performance is associated with higher fractional anisotropy (FA) or greater GM volume (Golestani and Pallier, 2007; Golestani et al., 2007; Bermudez et al., 2009; Della-Maggiore et al., 2009; Jäncke et al., 2009; Scholz et al., 2009; Foster and Zatorre, 2010a; Tomassini et al., 2011). Individual differences in structural measures reflect differences in the microstructural organization of tissue related to task performance. Greater FA, an index of fiber integrity, may represent a greater ability for neurons in connected regions to communicate (Fields, 2005, 2008); greater GM volume may indicate greater cell density and synaptic connections that could support enhanced information processing. However, some studies have found that better performance is associated with lower FA values (Tuch et al., 2005; Taubert et al., 2010). These somewhat counter-intuitive findings have been interpreted as potentially resulting from fibers that cross the identified tract. Analyses that could assess the contribution of crossing fibers to FA values have typically not been conducted. FA values in WM are affected by factors such as axon myelination, diameter, and packing density (Beaulieu, 2002; Alexander et al., 2007), but may also be influenced by the presence of crossing fibers (Douaud et al., 2009, 2011; Jbabdi et al., 2010). FA values in one fiber population can be affected by the relative strength of a second crossing fiber population in the same region. One way to assess the contribution of crossing fibers to FA is by assessing the differential contributions axial and radial diffusivity. However, because axial and radial diffusivity are defined relative to the axis of greatest diffusivity, rather than to particular tracts, their interpretation is non-trivial in a complex human brain with multiple fiber crossings (Jbabdi et al., 2010; Douaud et al., 2011). Therefore, fiber tractography should also be used to determine the underlying tract orientation in addition to clarifying FA correlations and/or differences by analysing axial and radial diffusivity.

While most neuroimaging studies examine task performance after a single day of training, the current study examined skilled performance and improvement after 5 days of practice. By combining behavioral data with cross-sectional DTI and T1 structural images obtained on the final day, we can identify the structural correlates of skilled motor performance and compare them with the brain regions functionally responsible for learning and performance on the same task. The results of our previous fMRI experiment showed that though most motor-related regions decreased in activity with learning, there were performance-related increases in specific regions including primary motor cortex, cerebellar lobule HVIIIa/VIIb, and superior parietal lobule (PLs) (Steele and Penhune, 2010). Therefore, in the current study we hypothesized that motor performance would be positively correlated with FA and GM volume in the regions functionally implicated in this task: motor cortex, cerebellum, and PLs. The secondary goal of this study was to more fully describe the contributions of axial and radial diffusivity to our FA findings and discuss them within the context of underlying tract organization defined by DTI tractography.

## Materials and methods

### Participants

The participants in this study were those tested in a previously published fMRI study (Steele and Penhune, 2010). The sample consisted of 13 participants (five female) between the ages of 18 and 27 (*M* = 22.4; *SD* = 2.9 years) who gave written informed consent. All were right handed [assessed using a handedness questionnaire adapted from Crovitz and Zener (1962)], neurologically normal, and had less than 3 years of musical experience [assessed using the Index of Musical Training and Experience; (Penhune et al., 1999)]. The experimental protocol was approved by the McGill University MNH/I Research Ethics Board and the Concordia University Human Research Ethics Committee.

### Task, stimuli, and procedure

The temporal motor sequence task (TMST) used in this experiment requires participants to reproduce a temporally complex sequence of finger taps in synchrony with a visual stimulus. This task can be used to detect both long- and short-term changes in performance and brain activity (Penhune and Doyon, 2005; Savion-Lemieux and Penhune, 2005; Steele and Penhune, 2010). Performance on this task can be separated into two components: (1) accuracy—the order of short and long key-presses in the sequence; and (2) synchronization—the precise timing of movements. A detailed description of the task, stimuli, and procedure is presented in a previously published functional imaging study (Steele and Penhune, 2010). In brief, participants learned to press and release a mouse button in synchrony with the onset and offset of a visually-presented sequence of 10 elements [5 (S)hort—300 ms; 5 (L)ong—600 ms; interstimulus interval—300 ms]. Each element was presented on screen for the specified duration as a large colored block—participants were instructed to press the mouse button when the block appeared and release when it disappeared. Five long and five short elements were arranged to create a sequence corresponding to a non-standard musical rhythm that is difficult to learn (the learning sequence—LRN: S L L S L S S L S L), a simple control sequence of five long followed by five short (L L L L L S S S S S), and a control sequence that was only observed. Four sequences of each condition were combined to create 40 s blocks. Four blocks of each condition were pseudorandomly arranged and interleaved with three 40 s blocks of rest to create a single training run. Participants were trained on the stimuli and taught LRN on the beginning of the first day and practised it for three runs of four blocks (16 trials) per day over 5 consecutive days, for a total of 240 trials. The current study focused on the relationship between the slope of improvement and final performance on LRN and cross-sectional structural imaging data acquired on the final day of training. T1 and diffusion-weighted images (DWI) were acquired with an eight-channel head coil in a Siemens Trio 3T MRI scanner on the final day of practice (T1–TR = 23 ms, TE = 7.4 ms, FOV = 256 mm, flip angle = 30°, 1 × 1 × 2 mm; DWI–3 runs of 32 directions, TR = 5000 ms, TE = 104 ms, FOV = 220 mm, *b* = 1000 s/mm^2^, 1.7 × 1.7 × 5 mm, five *b* = 0 images per run).

### Data analysis

#### Behavioral

Motor sequence skill was assessed with two measures of performance for each practice run: percent correct (PCOR)—the percentage of correctly produced long and short key-presses within the sequence, a measure of the accurate production of elements within the sequence, and percent synchronization (PSYN)—a measure of the synchronization of key-press responses with visual stimuli. Means (M) and standard deviations (SD) for short and long elements were calculated based on individuals' performance on training trials at the beginning of each day. PCOR was then defined as the percentage of key-press responses that were initiated between 300 ms before the stimulus and the end of the stimulus and had key-press duration of less than M + 2SD (for short elements) or greater than M - 2 SD (for long elements). PSYN was defined as the sum of the absolute lag between the onset and offset of the stimulus and the onset and offset of the response, divided by the actual stimulus element duration (Steele and Penhune, 2010). As this calculation results in values that are smaller for better performance, scores were subtracted from 100 to obtain a score that increased with performance. A score of 100% on PCOR represents perfect knowledge of the ordering of elements within the sequence. A score of 100% on PSYN indicates that the key-press and release response exactly matched the onset and offset of the visual stimuli.

For the purposes of this study two measures were used: final performance—PCOR and PSYN for the last run of training on Day 5; and slope of improvement—*r*-value of the best fit linear regression line passing through participants' PCOR and PSYN run averages for the 15 runs of the experiment (PCORslp, PSYNslp). Both measures index performance potential (how proficient you can become and how quickly that level can be attained) that we reasoned may be represented within the structure of the brain (Tomassini et al., 2011). Final PCOR, PSYN, PCORslp, and PSYNslp were then correlated with imaging measures as described below.

#### Diffusion imaging

All imaging data were analysed using the FMRIB Software Library (FSL 4.1.5) (Smith et al., 2004). Diffusion images from three diffusion runs were concatenated, corrected for eddy current, and averaged. The FMRIB's Diffusion Toolbox (FDT) was used to create voxelwise maps of diffusion parameters including FA and the eigenvalues of the diffusion tensor. Images were then analysed using FSL's tract-based spatial statistics (TBSS) (Smith et al., 2006) which first requires images to be non-linearly aligned to the FMRIB58_FA standard space template. The mean FA image was calculated and thinned to produce the study-specific FA skeleton—which represents the centers of all tracts common to all participants. FA data were then projected onto individual FA skeletons that were subsequently used in group permutation-based non-parametric statistical analyses. The mean FA skeleton was thresholded at FA > 0.25 to limit analyses to regions where major tracts existed in all individuals.

To determine the fiber regions that are important for skilled performance on this task, FA was correlated with final performance and slope measures for each participant with age as a covariate of no interest. Regions where FA was found to correlate significantly with performance were further investigated by assessing axial and radial diffusivity values. Whole-brain axial and radial diffusivity images were registered to the standard space using each individual's non-linear warp field (obtained from the FA image registration) and projected onto the mean FA skeleton. Regions identified in the FA correlational analysis were used to extract axial and radial diffusivity values from the same skeleton regions in all individuals. Partial correlation analyses, with age as a covariate of no interest, were then used to identify relationships between variables.

Probabilistic tractography was used to better characterize the directions of fiber tracts in regions of interest. This allows the interpretation of diffusion measures within the context of the underlying fiber tract organization. Significant voxels from the FA analysis were converted into a binary mask in each individual's 1 mm isotropic transformed diffusion space and then used to seed probabilistic tractography. Two different tractography analyses were conducted: one with target masks placed superiorly and inferiorly along the putative corticospinal tract (probable CST; inclusion planar regions at *z* = 54, 6, −11; exclusion at *x* = ±42 *y* = 43), the other with target masks placed laterally, anteriorly, and posteriorly to capture the association fibers/probable superior longitudinal fasciculus (probable SLF; inclusion planar regions at *x* = ±35, ±47 *y* = 42, −50; exclusion regions identical to CST inclusion). An additional exclusion plane was placed at *x* = 0 for both fiber populations. Both fiber directions were randomly sampled 10,000 times for each voxel in the seed mask. Each fiber population was averaged across participants and thresholded at 10% of the maximum particle number to obtain anatomically plausible tracts. This analysis produces delineations of the fiber tracts passing through the mask region, and can be used to visually differentiate the different fiber populations.

#### Voxel-based morphometry

To assess individual differences in GM volume that were related to task performance, T1 images were analysed with the VBM tools in FSL (Douaud et al., 2007). Images were brain extracted (Smith, 2002), then segmented by tissue type to produce 3D GM partial volume images (Zhang et al., 2001). Each image was first aligned to the MNI152 template brain with an affine transform (Jenkinson et al., 2002). A study-specific GM template was generated by averaging all linearly aligned GM images. The group mean GM image was used as the target for non-linear registration of the original native space GM images using a b-spline representation of the registration warp field (Rueckert et al., 1999). The resulting non-linearly aligned GM images were smoothed with a Gaussian kernel of σ = 4 (~9.4 mm) prior to statistical analyses. Whole-brain GM volume values were correlated with behavioral measures to identify cortical regions responsible for skilled performance and rate of improvement on this task.

Statistical analyses of FA and VBM data were conducted using FSL's randomize with 5000 permutations and threshold-free cluster enhancement (Smith and Nichols, 2009). All analyses were controlled for the effects of age (entered as a covariate of no interest) and results were considered significant at *p* < 0.05, corrected for multiple comparisons. Analyses resulting in significant correlations were rerun while controlling for both age and gender to confirm that the unequal number of males and females in this sample did not bias the results.

## Results

### Behavioral

**Figure 1:**
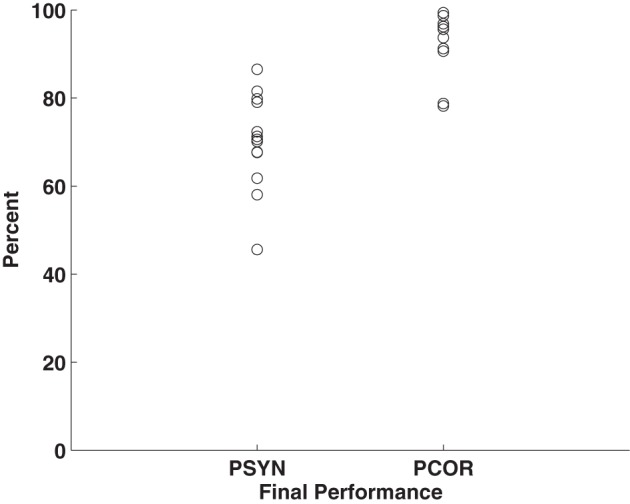
**Plot of individuals' final performance on PCOR and PSYN.** Points represent individual mean scores for the final run of Day 5.

Subjects were trained to produce accurate and synchronized button presses in response to a 10 element visually presented motor sequence across 5 days of practice. Within-subjects ANOVAs revealed that PCOR, the more explicit sequence ordering measure, improved significantly over the course of the experiment [PCOR: *F*_(4, 48)_ = 9.80, *p* < 0.01, η^2^ = 0.45] while PSYN, the more procedural sensorimotor integration measure, showed a statistical trend toward improvement on LRN (*p* = 0.10) (see Steele and Penhune, 2010 for further details). Final performance scores indicated that participants were able to perform well by the end of the experiment (PCOR: *M* = 93.13, *SD* = 7.06; PSYN: *M* = 70.16, *SD* = 10.84). Final PCOR performance was very high while final PSYN performance was lower and had greater variability across participants. Figure 1 plots each individual's PCOR and PSYN scores from the final run of practice—illustrating the inter-individual variability in behavioral performance. PCORslp (*M* = 0.80, *SD* = 0.82) and PSYNslp (*M* = 0.5, *SD* = 0.96) measures indicated that participants, on average, improved over the course of the experiment. Individual final PCOR and PSYN, PCORslp, and PSYNslp values were used in subsequent correlational analyses to explore the relationship between motor performance and brain structure.

### Diffusion measures

#### FA: behavioral correlation

**Figure 2:**
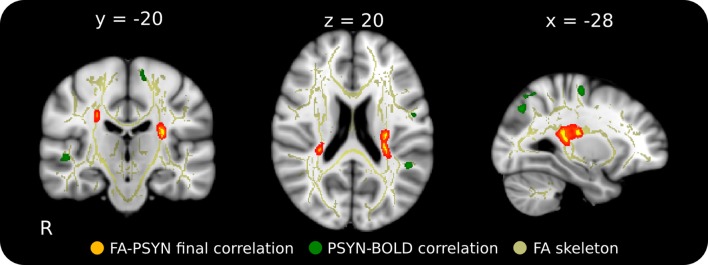
**Relationship between final synchronization performance and FA in WM underlying motor cortex in the context of regions functionally involved in this task.** This negative correlation suggests that those with greater performance have lower FA in these regions that correspond well to areas functionally implicated in the task. Red-Yellow: correlation between FA and final synchronization (*p* < 0.05, corrected for multiple comparisons); Green: functional correlation between synchronization performance and BOLD across the 5 days of the experiment (*p* < 0.001, cluster corrected); Yellow: mean FA skeleton (FA > 0.25). Significant voxels in the FA skeleton were thickened and overlaid on the ICBM 152 T1 for display.

FA was correlated with behavior to identify individual differences in WM integrity related to performance. Final PSYN was found to correlate negatively with FA within bilateral CST, such that participants with greater final synchronization performance had lower FA in these clusters. In the left hemisphere, one cluster was located directly below the hand area of the primary motor cortex (M1) (Yousry et al., 1997) and the other was located more inferiorly in the CST and extended into the temporal/parietal junction. The significant clusters in the right hemisphere were located in approximately the same regions as those in the left, though they were smaller. Figure 2 shows the regions of the FA skeleton negatively correlated with final PSYN performance overlaid with the regions where functional activity was positively correlated with PSYN performance (see Table 1 for a list of peak voxels and their locations). Final PCOR showed a similar relationship with FA in the same region of the left hemisphere as final PSYN, though this relationship was not significant after correcting for multiple comparisons. These findings indicate that the relationship between FA in this region and the task is a general one, rather than specific to a particular hemisphere or component of performance. A subsequent analysis including gender as an additional covariate of no interest found the same pattern of results as reported above: the cluster with peak voxel at −27, −30, 16 remained significantly correlated with Final PSYN and the remaining clusters dropped below significance to *p* = 0.07, fully corrected for multiple comparisons. There were no statistically significant correlations between PCORslp/PSYNslp and FA.

**Table 1:** **Coordinates and peak *t*-statistics for significant correlations**.

	**Location**	**Peak *t*-stat**	***x***	***y***	***z***
FA—final PSYN	L CST/SLF	−6.46	−27	−30	16
		−4.36	−28	−20	19
	R CST/SLF	−6.34	31	−34	16
		−5.70	25	−22	31
		−3.99	20	−26	48
VBM—PSYN slope	Lobule V	12.38	−4	−58	−16
	R Lobule HVI	8.7	32	−48	−30

#### Axial and radial diffusivity

**Figure 3:**
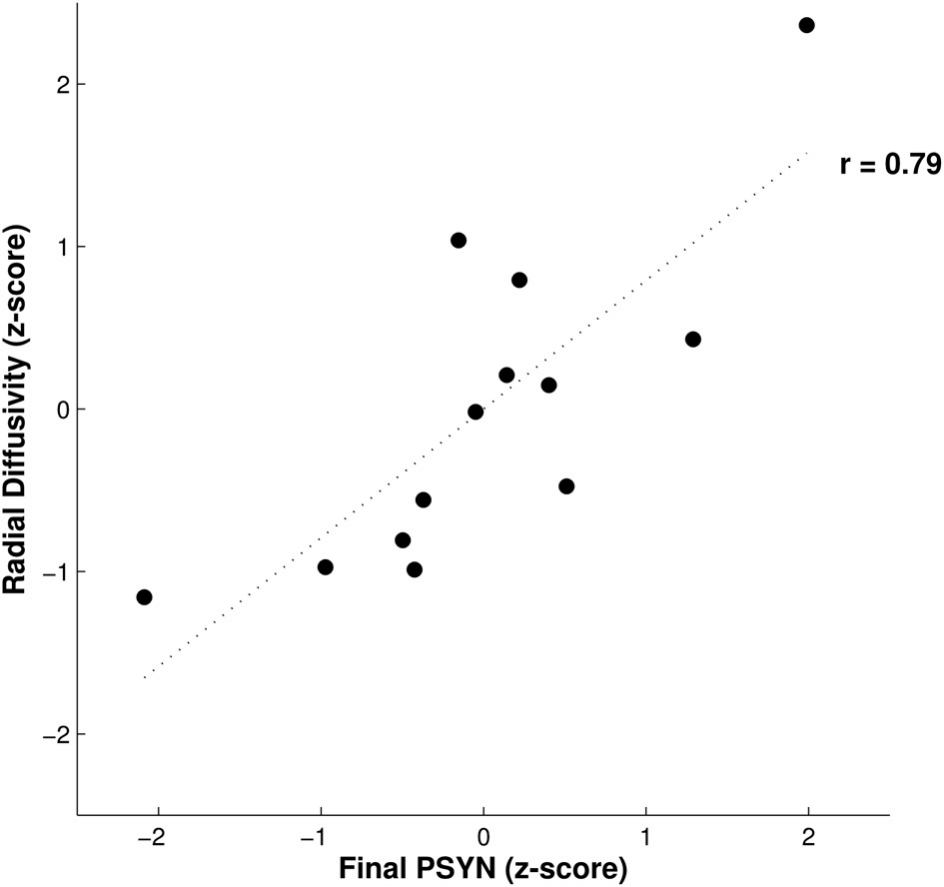
**Relationship between radial diffusivity and final performance on PSYN.** This plot depicts the partial correlation between final PSYN and radial diffusivity extracted from the significant correlation with FA in bilateral sensorimotor cortex (Figure 2) after the effects of age have been removed. Each value is a residual converted to a *z*-score, and represents individual scores. The dotted line represents the best fit linear regression line through the data (*r* = 0.79, *p* < 0.005).

Axial and radial diffusivity values were extracted from the identified region to investigate their contributions to the negative correlation between FA and final PSYN. Axial diffusivity is the diffusivity along the axis of greatest diffusion and radial diffusivity is the mean of diffusivity in the two perpendicular axes. Radial diffusivity was found to positively correlate with final PSYN (*r* = 0.79, *p* < 0.005) while axial diffusivity did not (*p* = 0.18). In addition, FA and radial diffusivity were negatively correlated (*r* = −0.91, *p* < 0.001). Figure 3 shows the partial correlation between radial diffusivity and final PSYN performance. The positive correlation between radial diffusivity and performance combined with the strong negative correlation between FA and radial diffusivity indicates that the observed negative relationship between FA and performance is driven by the positive relationship between radial diffusivity and performance.

#### Tractography

**Figure 4:**
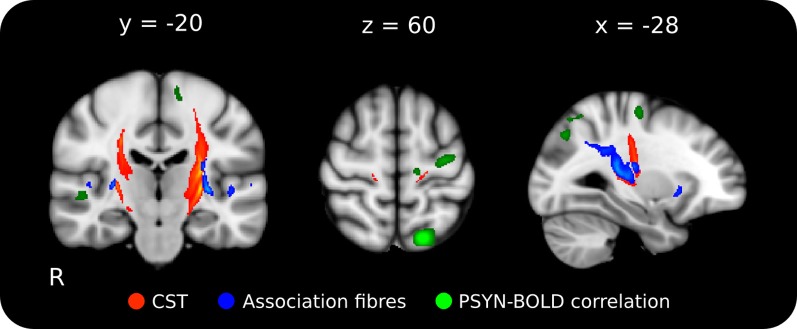
**Mean probabilistic tractography results for each target region, originating from the performance-FA seed mask.** Red-Yellow: tract resulting from the ascending/descending waypoint masks; Blue-Lightblue: tracts resulting from the lateral waypoint masks; Green: functional correlation between synchronization performance and BOLD across the 5 days of the experiment (*p* < 0.001, cluster corrected). The delineation between the ascending/descending fibers of the CST and the laterally projecting fibers can be clearly seen. Tractography was conducted on each individual, averaged, and thresholded at 10% of maximum for display. Lighter colors indicate higher particle count. The tractography seed mask contained all voxels in the skeleton that showed a significant negative correlation between FA and final PSYN performance in both hemispheres. Tracts have been overlaid on the ICBM 152 T1 average for display.

**Figure 5:**
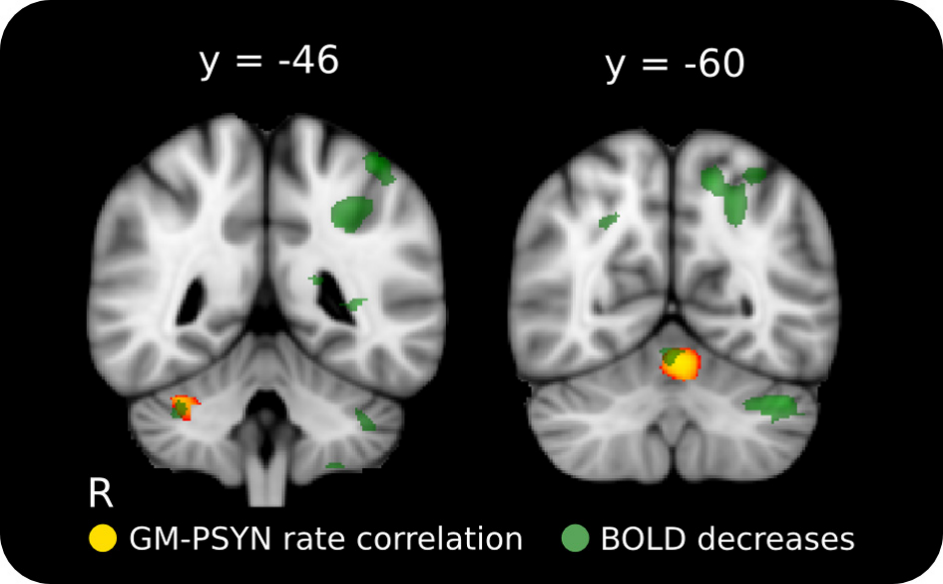
**Positive relationship between rate of improvement on synchronization and GM volume in cerebellar lobules HVI and V.** The cerebellar structures identified here correspond well with two regions where BOLD activity decreased across learning on this task. Red-Yellow: significant correlation between GM volume and rate of improvement (*p* < 0.05, corrected for multiple comparisons); Semi-Transparent Green: task-specific decrease in BOLD activity between Day 2 and Day 5 (*p* < 0.001, cluster corrected). Significant regions are overlaid on the ICBM 152 T1 average for display.

Probabilistic tractography was used to identify the tracts crossing the region identified in the FA-behavioral analysis to more precisely interpret the results of the FA, axial, and radial diffusivity analyses. Based on location, we expected that the clusters identified in the behavioral regression analyses could contain fibers not only from the CST but also from the SLF. To test this possibility, we performed probabilistic tractography on two combinations of target and exclusion masks designed to delineate ascending and descending (probable CST) from association fibers (probable SLF). Using the region where FA was found to significantly correlate with final PSYN as the seed (inclusion planar regions at *z* = 54, 6, −11; exclusion at *x* = ±42 *y* = 43), the ascending and descending tract extends superiorly to the sensorimotor cortex and inferiorly to the brainstem; this tract location is consistent with the CST (colored Red-Yellow in Figure 4) (Wakana et al., 2004) and the cortical target of its trajectory corresponds well with the motor cortical regions found to increase with improvements in PSYN (green in Figure 4) (Steele and Penhune, 2010). The tract identified with same seed and lateral, anterior, and posterior target masks (inclusion planar regions at *x* = ±35, ±47 *y* = 42, −50; exclusion regions identical to CST inclusion) is consistent with the course of the SLF: extending anteriorly to the frontal lobe along the external capsule, posteriorly across the superior part of the CST to the parietal lobe, and laterally to the auditory cortical regions of the temporal lobes (colored Blue-Lightblue in Figure 4) (Mori et al., 2002; Wakana et al., 2004; Makris et al., 2005). The tract termination points show remarkable agreement with the parietal and auditory cortical regions previously found to be involved in optimizing this component of the task (green in Figure 5) (Steele and Penhune, 2010). The excellent correspondence between the functionally-defined motor, parietal, and auditory cortical regions important for PSYN optimization and the tracts identified in this analysis underscore the importance of these regions and their connections in the optimization and performance of this task.

### Voxel-based morphometry and performance

To compliment the WM findings, we used VBM to examine regions of the GM that may contribute to the acquisition and performance of the TMST. PSYNslp was positively correlated with GM volume in right cerebellar lobules HVI and V (Schmahmann et al., 2000), two regions known to be specifically connected to the motor cortex (Figure 5, depicted in red to yellow) (Kelly and Strick, 2003; O'Reilly et al., 2010; Stoodley and Schmahmann, 2009). Refer to Table 1 for a list of peak voxels and their locations. Importantly, these regions showed significant learning-related decreases in blood-oxygenation-level-dependent (BOLD) signal between Day 2 and Day 5 in the functional study with the same participants (Figure 5, depicted in green) (Steele and Penhune, 2010, supplementary materials). An additional analysis including age and gender as covariates of no interest found the same two regions to be significantly correlated with PSYNslp, though at a reduced spatial extent. There were no significant correlations with any of the other measures.

## Discussion

The current study examined the relationship between individual differences in the ability to perform a motor task and structural brain measures collected on the final day of practice. Importantly, we compared these findings with the results of a previous functional brain imaging study in the same sample. Behavioral regression analyses found that better final synchronization performance was negatively correlated with FA bilaterally in fiber tracts underlying sensorimotor cortex, such that participants with lower FA showed better final performance. The direction of this relationship may appear counter-intuitive. However, radial diffusivity in this region was positively correlated with performance and multi-fiber tractography revealed that this region is an area of CST and SLF crossing fibers—meaning that the interpretation of FA in which bigger is better may not always apply. Functional imaging results with the same sample found positive relationships with synchronization performance in motor, parietal, and auditory cortical regions that correspond well with both identified tracts (Steele and Penhune, 2010). These findings raise the possibility that skilled performance on this task is associated with enhanced fiber integrity in the SLF. Enhanced fiber integrity in the SLF could result in reduced FA in regions where it crosses the CST. Additional VBM analyses revealed a positive relationship between rate of improvement and GM volume in right cerebellum that were co-localized with functional decreases observed in the fMRI data (Steele and Penhune, 2010), thus providing further evidence for the cerebellum's role in skilled motor performance.

Better final performance on the TMST was related to lower FA in the CST and SLF inferior to bilateral sensorimotor cortex, and this effect was mediated by a positive correlation with radial diffusivity (Figures 2, 3). Our tractography result confirmed that this region contained fibers from both the CST and SLF (Makris et al., 2005) (Figure 4). Although we were unable to detect tract-specific relationships—likely as a result of the non-isotropic voxel sizes used in the current study—we speculated that greater diffusivity along the course of the SLF in this region may be responsible for the observed positive correlation of performance with radial, rather than axial, diffusivity. Though increases/greater radial diffusivity has been linked to dysmyelination in uniformly oriented fiber populations (Pierpaoli et al., 2001; Sun et al., 2008), the presence of crossing fibers in this region makes interpretation more difficult (Jbabdi et al., 2010; Douaud et al., 2011). Therefore, we have hypothesized that radial diffusivity in part reflects the fiber integrity of the SLF, where the principle diffusion direction is typically oriented anterior-posterior. The possibility that the negative correlation between FA and skilled performance could be driven by variation in the SLF is an attractive one. The SLF connects parietal and auditory cortical regions functionally implicated in performance of this task and in an fMRI study with the same participants (Penhune and Doyon, 2002, 2005; Steele and Penhune, 2010). In support of our hypothesis, a previous study found a *positive* relationship between FA in the SLF and motor sequence learning (Tomassini et al., 2011). This finding is in a more anterior region of the SLF (*y* = −10) that would be unlikely to be influenced by crossing fibers from the CST. Also possibly consistent with our findings, a recent study showed that *non-musicians* had *greater* FA than musicians in bilateral CST regions similar to those observed in our results (Imfeld et al., 2009). The authors speculate that their counter-intuitive findings are due to increased axonal permeability due to long-term sensorimotor training in musicians, but do not consider the potential effect of crossing fibers. The overlap between the regions functionally implicated in improvement on the TMST and the tractography results presented here provides further evidence for the importance of the SLF in skilled motor sequence performance.

The results of behavioral regression analyses with VBM GM values showed that individual differences in cerebellar lobules HVI and V were related to the rate of improvement of synchronization on the TMST. These regions overlap with those that showed learning-related decreases in activity in the fMRI data from the same subjects (Figure 5). Co-localization of behaviorally-relevant structural differences and functional changes identified with independent analyses provides further evidence for the role of the cerebellum in motor tasks—a finding that is compatible with a proposed role for the cerebellum in processing error-related feedback (Ohyama et al., 2003). Crucially, lobules HVI and V are structurally and functionally connected to motor cortex (Kelly and Strick, 2003; O'Reilly et al., 2010; Stoodley and Schmahmann, 2009), show performance-related changes in functional activity during motor tasks (Penhune and Steele, 2012), and form part of a network of regions responsible for the optimization of motor behavior (Ramnani, 2006).

Studies identifying relationships between cerebellar GM volume and performance are rare, with only two that use non-expert populations (Tomassini et al., 2011; Kühn et al., 2012). Our results are in agreement with those of Kühn et al. (2012), who found that GM volume in lobule VI was related to fine motor control, and directly support those of Tomassini et al. (2011) who also identified a relationship between motor sequence performance and GM volume in lobule VI. Though the design of the current study did not allow us to address learning-related changes in GM volume, previous work has identified increases in cerebellar synapse number and glial cell volume as a result of practice and learning (Kleim et al., 2002, 2007). We hypothesise that the observed performance-related individual differences in GM are in part due to differences arising from previous training and experience. Thus, greater cell or synaptic density in the cerebellum may support enhanced information processing ability (and thus a faster rate of behavioral improvement) that is related to decreasing functional activity as performance improves.

The causes of inter-individual variability in brain structure are not fully understood, but likely include pre-existing genetic contributions and contributions from learning and the environment. The design of our study was not able to disentangle these affects. FA is affected by WM properties including axon myelination, diameter, and packing density. Differences in these properties could lead to the individual differences in performance observed in our study through pre-existing differences or training-induced changes in axon conduction velocity and synaptic synchronization (Fields, 2005, 2008), or density of innervation. Greater fiber integrity along the SLF would be consistent with the idea, proposed by Fields, that greater myelination observed in relation to performance may underlie enhancements in synchronized activity between task-relevant regions (Fields, 2005, 2011). Similar to WM measures, individual differences in GM volume could be influenced by multiple factors such as neuronal and glial cell density, synaptic density, vascular architecture, and cortical thickness. Though the physiological basis for GM volume differences in humans has not been fully explained, previous work has established the feasibility of identifying individual differences in brain structure that are related to: timed finger tapping (Ullén et al., 2008), performance on musical tasks (Foster and Zatorre, 2010b), bimanual coordination (Johansen-Berg et al., 2007), and learning of foreign language sounds (Golestani and Pallier, 2007; Golestani et al., 2007). This study identified regions where performance is related to brain structure but its design does not allow us to conclude whether the observed effects are due to previous experience, training, or a combination of the two. Our study comprised a brief training regime (5 days) followed by structural data acquisition on the final day. A number of studies have identified structural changes after multiple weeks of training (Draganski et al., 2004; Boyke et al., 2008; Scholz et al., 2009; Taubert et al., 2010), but others have also reported changes with short-term training (Landi et al., 2011), TMS (May et al., 2007), and drug intervention (Tost et al., 2010). The current study provides a link between skilled performance and brain structure in regions known to be functionally involved with task performance. With only a single timepoint we cannot comment on how the regions that we have identified may change as a result of practice; however, given the overlap with previous fMRI results, structural changes in the SLF and cerebellar lobules HVI and V may occur with training on similar motor sequence tasks. Future studies employing longitudinal methods and longer periods of training could be used to address these questions.

We have identified individual differences in performance that are related to brain structures important for motor sequence performance. There was a negative relationship between FA and performance in a region of the CST-SLF fiber crossing that may reflect greater fiber integrity in the SLF of skilled performers—and is consistent with the idea of enhanced communication/synchronization between regions functionally important for this task. Two regions of the cerebellum (lobules HVI and V) where GM volume is important for the speed at which sequence skill is acquired were also identified. Our multimodal cross-sectional individual differences design also illustrates the importance of considering multiple structural measures (GM volume, FA, diffusivities, tractography) within the context of functional results to help provide a more global interpretation of the processes involved in skilled motor sequence performance.

### Conflict of interest statement

The authors declare that the research was conducted in the absence of any commercial or financial relationships that could be construed as a potential conflict of interest.
